# If Time Is Brain Where Is the Improvement in Prehospital Time after Stroke?

**DOI:** 10.3389/fneur.2017.00617

**Published:** 2017-11-20

**Authors:** Jeremy N. Pulvers, John D. G. Watson

**Affiliations:** ^1^Sydney Adventist Hospital Clinical School, Sydney Medical School, The University of Sydney, Wahroonga, NSW, Australia

**Keywords:** stroke, prehospital delay, thrombolysis, tissue plasminogen activator, emergency medical services

## Abstract

Despite the availability of thrombolytic and endovascular therapy for acute ischemic stroke, many patients are ineligible due to delayed hospital arrival. The identification of factors related to either early or delayed hospital arrival may reveal potential targets of intervention to reduce prehospital delay and improve access to time-critical thrombolysis and clot retrieval therapy. Here, we have reviewed studies reporting on factors associated with either early or delayed hospital arrival after stroke, together with an analysis of stroke onset to hospital arrival times. Much effort in the stroke treatment community has been devoted to reducing door-to-needle times with encouraging improvements. However, this review has revealed that the median onset-to-door times and the percentage of stroke patients arriving before the logistically critical 3 h have shown little improvement in the past two decades. Major factors affecting prehospital time were related to emergency medical pathways, stroke symptomatology, patient and bystander behavior, patient health characteristics, and stroke treatment awareness. Interventions addressing these factors may prove effective in reducing prehospital delay, allowing prompt diagnosis, which in turn may increase the rates and/or efficacy of acute treatments such as thrombolysis and clot retrieval therapy and thereby improve stroke outcomes.

## Introduction

The “*time is brain*” concept introduced more than two decades ago (1) encapsulates the crucial importance of time in treating acute stroke. This has become more pertinent since the advent of thrombolysis treatment using tissue plasminogen activator (2, 3) and endovascular therapy (4). Regarding thrombolysis, benefit has been shown for initiating treatment up to 4.5 h after acute stroke onset (5, 6). A major obstacle to their use however is a long onset-to-door time (from stroke symptom onset or time last known well to hospital arrival), which in general is the largest component of total onset-to-needle time (from stroke onset to thrombolysis) (7, 8).

Previous reviews of prehospital delay have shown little improvement in onset-to-door times over the years (7, 8). Much effort to reduce door-to-needle times have led to remarkable improvements (9); however, these efforts on reducing in-hospital delay are diminished by the minimal improvements in prehospital delay. The battle to increase thrombolysis rates will remain futile unless significant improvements are seen in reducing onset-to-door times after acute stroke (8, 10).

Reducing the time to hospital arrival is crucial for prompt diagnosis and timely delivery of therapies such as thrombolysis and clot retrieval. However, analysis of trial data has not consistently shown a relationship between time to treatment and better outcomes (11–13). Nevertheless, early arrival will naturally lead to a higher proportion of acute strokes arriving within the therapeutic time windows, conferring improved outcomes on a higher proportion of patients, regardless of whether there is increased benefit earlier in the 4.5-h thrombolysis time window. A study analyzing the baseline penumbra volume, baseline ischemic core volume, and the penumbra salvaged from infarction after thrombolysis, showed that greater penumbral salvage had the greatest effect on disability-free life, rather than onset to treatment time (14). However, this does not negate the importance of early presentation in this context, as it allows more time for prompt clinical and imaging assessment. Moreover, earlier presentation should allow for a more extensive evaluation of stroke mimics and potential misdiagnoses (15–17), within the time window of eligibility for acute stroke therapies.

The identification of factors associated with early or delayed hospital arrival after stroke is of crucial importance in improving thrombolysis rates (10, 18) and by extrapolation the rates of other acute interventions. We therefore conducted a review of studies that analyzed factors associated with either early or delayed hospital arrival after stroke, with the aim of identifying modifiable targets of interventions in reducing prehospital delay. Knowledge of these factors may be helpful in reducing onset-to-door times, and thus increase the implementation rates of acute stroke therapies.

## Review Methods

A search of MEDLINE was performed *via* Ovid (http://ovidsp.ovid.com) using a previously published search strategy (7, 8) between 2008 to the access date of November 1st 2016. For the years prior to 2008, references of previous reviews were examined (7, 8, 10, 18, 19). The same search strategy was also used in Embase *via* Ovid excluding MEDLINE journals but with no limit on publication year. Studies not published in English, review articles, and Letters to the Editor were excluded. The following were also excluded: studies focusing solely on transient ischemic attacks (TIA); studies that reported on hospital arrival times but did not analyze factors associated with early or delayed arrival; studies on decision delay after stroke; studies on delay to alerting medical services or delay to first medical contact, and delay to admission to stroke unit; and studies on factors associated with Emergency Medical Services (EMS) use.

115 studies, published between 1990 (20) and 2016 (21) reporting on data acquired between 1985 (20) and 2013 (22), were identified that focused primarily on analyzing factors associated with early or delayed hospital arrival after stroke. From these studies, factors significantly associated with early or delayed hospital arrival were extracted and are listed in Table 1. Factors from studies that did not describe any statistical analyses were excluded (22–29). Factor data were excluded from one study which defined early arrival as before 24 h (20).

**Table 1:** Factors associated with early and delayed hospital arrival after stroke.

Factors associated with early presentation
Emergency Medical Services admission (40) (30–69)
Severe stroke (NIHSS and equivalent) (26) (38, 43, 45, 46, 50, 54, 56, 58, 63–65, 69–83)
Hemorrhagic stroke (10) (57, 59, 61, 66, 84–89)
Consciousness: lowered, disturbed, lost (9) (41, 49, 61, 81, 85, 90–93)
History of stroke or TIA (7) (41, 62, 73, 81, 94–96)
History of atrial fibrillation, cardiac arrhythmia (7) (43, 56, 61, 62, 67, 97, 98)
Attributing symptoms to stroke (7) (53, 55, 69, 92, 98–100)
CAD, IHD, prior myocardial infarction (6) (56, 59, 61, 62, 96, 101)
Perception of severity, urgency (6) (32, 43, 47, 52, 53, 100)
Speech disturbance, aphasia (6) (41, 44, 52, 57, 102, 103)
911 (or equivalent) called first or early (6) (32, 99, 104–107)
Bystander response (5) (32, 47, 49, 58, 99)
Not living alone (4) (33, 39, 60, 82)
Higher education level (4) (43, 60, 77, 101)
TIA (4) (43, 57, 89, 100)
Increasing disability (4) (71, 78, 86, 88)
Daytime onset (4) (70, 79, 86, 108)
Sudden onset of symptoms (3) (39, 71, 99)
Reduced GCS (3) (45, 78, 95)
Knowledge of thrombolysis (3) (53, 58, 68)
Cardioembolic stroke (3) (89, 109, 110)
Motor impairment (3) (41, 71, 111)
White race/ethnicity (USA) (3) (33, 48, 62)
Directly reaching hospital (3) (89, 101, 102)
**Factors associated with delayed presentation**
Primary care facility (GP) visited first (14) (34, 59, 61, 68, 85, 90, 112–119)
Referral from other hospital (10) (49, 58, 66, 74, 92, 97, 115, 117, 120, 121)
Living alone (9) (43, 59, 60, 66, 68, 71, 94, 95, 122)
Stroke in the evening or night (8) (40, 59, 66, 82, 85, 92, 104, 122)
Diabetes mellitus (7) (52, 55, 56, 61, 62, 67, 92)
Private transport to hospital (6) (60, 63, 97, 113, 119, 121)
Black race/ethnicity (USA, UK) (5) (54, 56, 82, 123, 124)
Lacunar stroke, small vessel stroke (5) (46, 90, 95, 96, 109)
Mild neurological symptoms (5) (34, 59, 63, 94, 113)
Symptoms not taken seriously, low threat perception (4) (43, 59, 114, 117)
Awakening with symptoms (3) (35, 125, 126)
Symptom onset at home (3) (61, 97, 104)
Regular drinker, history of alcohol abuse (3) (61, 79, 96)
Worsening symptoms compared to onset (3) (49, 97, 121)

*Factors significantly associated (*P* < 0.05) with early hospital arrival after stroke are shown. Factors were included in this table if they were reported as significant in three or more studies. Factors independently associated with early or delayed arrival (multivariate analysis) were included in the list; however, for studies that performed univariate analyses only, these factors are also listed*.

*The first number in parentheses indicates the number of studies canvassing each factor, followed by the references*.

*NIHSS, National Institutes of Health Stroke Scale; TIA, transient ischemic attack; CAD, coronary artery disease; IHD, ischemic heart disease; GCS, Glasgow Coma Scale; GP, general practitioner*.

Median onset-to-door times, and the cumulative percentage of patients arriving at hospital within: 1, 2, 3, 6, and/or 24 h (majority of studies described data for these time intervals), were collected when available. When median times were lacking in a study, but a percent arriving before a given hour was 50% (±1%), this time was used as the median arrival time. Similarly, when median times fell exactly on the time intervals above, then 50% was added to the data as the cumulative percentage arriving before that time. When time data were subdivided into certain population subgroups, these were excluded. When time data were obtained over a range of years, the mean of the years was used (8). Time data were excluded from one study that only included patients that received thrombolysis (80). Inclusion criteria based on stroke subtype varied widely (7, 8, 18), for example: stroke and stroke-like symptoms (32), ischemic only (58), ischemic and hemorrhagic (127), stroke excluding subarachnoid hemorrhage (50), intracerebral hemorrhage only (78), and some included TIA (43). Other notable methodological variations were (i) time interval defining early versus delayed arrival; (ii) whether a cutoff was used to exclude prehospital time data from cases of prolonged (e.g., >24 h) delay; and (iii) how prehospital time was defined in cases of patients awakening with stroke (7, 8, 18).

## Time from Symptom Onset to Hospital Arrival: Trends Over two Decades

**Figure 1:**
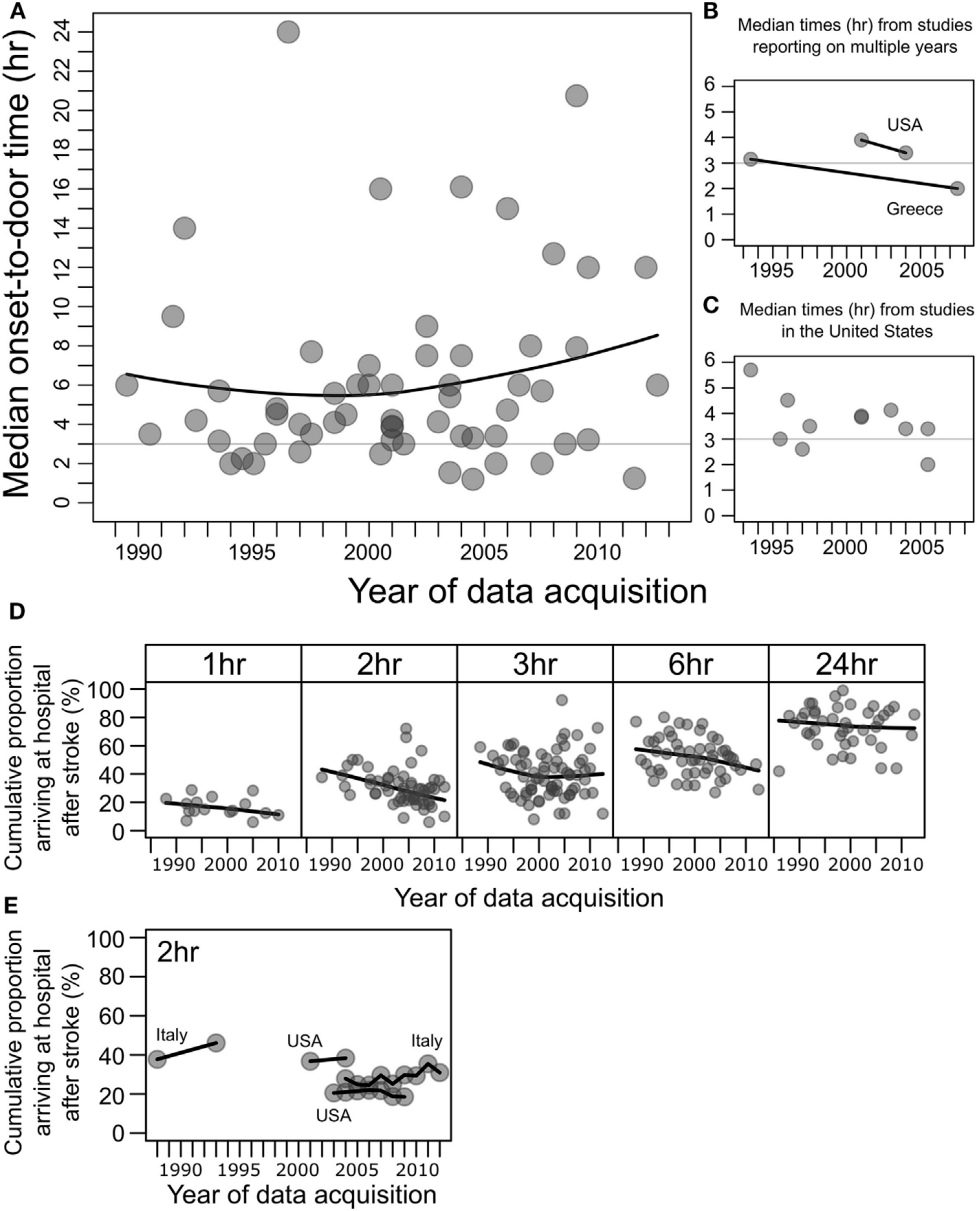
Median onset-to-door times after stroke and percentages of patients arriving to hospital after stroke at 1, 2, 3, 6, and 24 h. **(A)** Data points represent median onset-to-door times (hours) of stroke patients plotted against the year/s of data acquisition, in studies of factors associated with hospital arrival times after stroke, from 58 studies. For studies conducted over multiple years, the mean of the years was taken (8). Black line shows the local polynomial regression (LOESS), and the horizontal gray line indicates 3 h. **(B)** Median onset-to-door times (hours) from two studies that reported data for multiple years, from the United States (USA) (54) and Greece (59). Black lines connect data from the same study. **(C)** Subset of median onset-to-door time data in panel **(A)** showing studies from the United States (31–33, 35, 36, 44, 48, 53, 54, 73), excluding one outlier of median 16 h in 2000–2001 (128). **(D)** The cumulative percentages of patients arriving to hospital after stroke, at 1, 2, 3, 6, and 24 h after onset. Data points represent percentages from individual studies plotted against year/s of data acquisition. Black line shows the local polynomial regression (LOESS). An improvement in prehospital delay over the years would manifest as an upwards curve within each box, which is not seen. **(E)** Subset of the cumulative percentage of patients arriving before 2 h from studies that reported on data for multiple years from Italy [1986–1990 to 1991–1995 (71); 2004–2012 (83)] and the United States [2001–2004 (54), 2003–2009 (62)].

Within the 115 studies reviewed here, 58 studies from 26 countries contained median onset-to-door times and the year/s of data acquisition (Figure 1A). The key and perhaps unexpected result is that onset-to-door time over the years has essentially remained unchanged, as previously reported for data up to 2006 (7, 8). The majority of studies reported a median onset-to-door time well beyond 3 h, which when taking door-to-needle time in consideration, prohibits the effective and timely commencement of thrombolytic therapy. Only two studies (54, 59) showed median onset-to-door times from different years, which exhibited only modest improvements (Figure 1B). Eleven studies originating from the United States, the country with the most studies available for secular trend comparison, showed no meaningful improvement overall (Figure 1C). An analysis of onset-to-door time data from the Get With The Guidelines program between 2003 and 2009 (62) showed essentially no improvement (Figure 1E).

Within the 115 studies reviewed here, 100 studies contained data on the cumulative percentage of stroke patients arriving at hospital before at least one of the following time intervals: 1, 2, 3, 6, and/or 24 h, and also the year/s of data acquisition (Figure 1D). The majority of patients failed to arrive before 3 h, and the local regression shows no improvement over the two decades. Four studies (54, 62, 71, 83) showed percentages of patients arriving before 2 h from different years, and these essentially showed no improvement overall (Figure 1E).

Despite the advent of thrombolytic therapy for acute ischemic stroke in the late 1990s (2, 3), the majority of patients in the majority of locations around the world failed to arrive at hospital before 3 h (7, 8). When taking door-to-needle time into consideration, which although improving (9) is commonly in excess of 1 h (7, 8), a 3-h onset-to-door time would generally be the maximum delay possible to meet a 4.5-h onset-to-needle time target for thrombolysis (5, 6). Improvements in prehospital time have been stagnant, and it remains the largest component of total onset-to-needle time (7, 8). A dramatic example of this is a study from Greece analyzing 16 years of onset-to-emergency room presentation (prehospital time) and emergency room to completion of CT times (a component of in-hospital time), which showed a more than 10 h decrease in in-hospital time (median of 12.34 to 1.05 h), whereas prehospital time was reduced only by about 1 h (median of 3.15 to 2.0 h) (59).

## Factors Associated with Early and Delayed Hospital Arrival after Stroke

From the studies reviewed here, factors associated with either early or delayed arrival after stroke were extracted (Table 1). Patient age and sex were associated in different studies with both early and delayed arrival and are discussed separately.

Hospital arrival by EMS was the factor most frequently associated with early hospital arrival after stroke, with 40 reporting studies. Severe stroke was the second most frequent factor, as measured by the National Institutes of Health Stroke Scale (NIHSS) or other scales. Other factors associated with early arrival were related to stroke symptomatology, stroke subtype, comorbidities, patient and/or bystander behavior or perception at stroke onset, and timing of stroke onset.

The top three factors associated with delayed arrival were if a general practitioner (GP) or primary care facility was visited first, referral from another hospital, and living alone.

## The Key to Early Hospital Arrival: EMS

Hospital admission *via* EMS was by far the most frequently associated factor with early arrival, with the converse non-EMS use, appearing high in the list of factors associated with delay. A review of surveys on the knowledge of what action to take upon stroke symptom onset has shown that, although the majority stated calling EMS, a sizable proportion responded contacting their GP (129). It is essential that educational programs further emphasize contacting EMS immediately upon stroke onset (10, 18).

Three factors frequently associated with delayed arrival were closely related: primary care facility visited first, referral from another hospital, and private transport to hospital. These reveal the importance of patient and/or bystander factors, such as misjudgment at symptom onset or poor awareness of stroke symptoms and emergency pathways, and further stress the necessity of raising the awareness of the variability of stroke symptoms (18). This is exemplified by the fact that mild neurological symptoms, which may be misinterpreted as general malaise and thus minimized in seriousness by patients and bystanders (34), were significantly associated with delayed arrival.

One study that analyzed factors associated with EMS-use after stroke found that of the cases where EMS was activated, only 4.3% of calls were made by the patient compared to 60.1% by family members, stressing the importance of targeting potential latent bystanders (family, caregivers, and coworkers) in educational programs (130). A study of a community and professional behavioral intervention program on stroke identification and management showed an increase in thrombolysis rates between the intervention and comparison group, however not in delay time (131). EMS use is known to have additional benefits beyond shortening of prehospital time. Studies have shown that, due to hospital pre-notification (132), EMS use is associated with prompter evaluation by imaging, shorter door-to-needle times, and increased thrombolysis rates (133). Therefore, the nature of transport to hospital (EMS versus private transport) has an added benefit to in-hospital stroke care beyond the simple shortening of prehospital time.

## Stroke Subtype, Symptomatology, and comorbidities

Severe stroke was a major factor associated with early arrival, which is to be expected by its debilitating symptomatology, naturally raising a sense of urgency in the patient or bystander. Interestingly, a history of cardiac arrhythmia or atrial fibrillation (AF) was associated with early arrival. Patients with AF are known generally to present with more severe strokes (134) which may be a contributing factor to early presentation. Patients may also have a latent sense of urgency to present to hospital with new symptoms, because of their known cardiac condition (43), or have a raised awareness of stroke symptoms, with AF being a major stroke risk factor (135).

Diabetes mellitus was associated with delayed arrival after stroke in multiple studies. This may be due to patients or bystanders misinterpreting symptoms as hypoglycemia (92). Moreover, diabetics versus non-diabetics were shown to more likely present with lacunar and ischemic strokes with a lower rate of hemorrhagic strokes (136, 137). Patients with lacunar strokes show delayed presentation and hemorrhagic strokes present earlier (Table 1), and thus the delay in stroke patients with diabetes may be due to differences in stroke subtype or symptomatology rather than diabetes *per se*. More investigation is required as this may be a promising target for intervention. A number of other vascular risk factors were also associated with delayed arrival (52) such as smoking and hypertension (56, 62).

## Perceptual and Behavioral Factors

Perceptual and behavioral factors (99) such as symptoms not taken seriously and low threat perception were also associated with delayed arrival. Past research on stroke knowledge has shown that having stroke risk factors in general does not contribute to an increase in stroke knowledge (129, 138), which further stresses the importance of improving knowledge through public awareness campaigns (18, 139, 140). Such campaigns must target those with stroke risk factors (141), and also be tailored to target minority populations (142). However, the fact that a personal history of stroke or TIA was significantly associated with early arrival points to the effectiveness of the sense of urgency or awareness that comes about by a first-hand experience of cerebrovascular disease in reducing onset-to-door time (71, 94). Family history of stroke was also associated with early arrival (71, 114), and this has also been shown to be an independent predictor of knowing at least one stroke risk factor (143). Promisingly, the knowledge of thrombolysis treatment by patients was associated with early arrival (Table 1).

## Time to Hospital Arrival: Male Versus Female and Patient Age

Depending on the study, female compared with male patients were associated with both early (39, 54) and delayed arrival (47, 56, 62, 99, 144, 145). Many factors may contribute to this difference, including comorbidities, prestroke disability (145, 146) and whether they live alone (147, 148). Differences in stroke subtype and symptomatology between men and women may underlie differences in arrival time (148–150), and moreover it is important to consider disparities in stroke outcomes not just arrival times (151).

There is no conclusive relationship between patient age and prehospital time. Studies utilized various methods for analyzing the effect of age. In short, being younger was associated both with early (63, 83, 96) and delayed (57, 91, 126) presentation, and similarly older patients were associated with both early (37, 43, 59, 77, 114) and delayed (56, 57, 62, 74, 83, 121) presentation. There may be a lack of urgency in younger patients with stroke (152), and symptoms exhibited by older patients may be more readily interpreted as stroke and perceived as an emergency (43). Interestingly, a review on studies of stroke knowledge reported that stroke knowledge is generally lowest in the young (18–25 years) and the elderly (≥80 years) (18).

## Interaction Between Onset-to-Door and Door-to Needle Time: A Virtuous Cycle

Numerous studies have reported on the phenomenon of an inverse correlation between onset-to-door time and door-to-needle time (153–156). This is thought to be due to physicians treating more urgently those patients who are approaching the end of the thrombolysis time window than patients with earlier presentations (156). Door-to-needle time may be taken as a surrogate global measure of health service-controlled stroke care quality, and given that a personal or family history of cerebrovascular disease and knowledge of thrombolysis are factors associated with early presentation, a scenario can be imagined where improvements in door-to-needle times may, in turn, lead to an improvement in onset-to-door times, supported by the fact that family and friends are a source of stroke knowledge and awareness (129, 138). As patients further recognize the benefits of available acute therapy for stroke, and if in-hospital pathways can be improved so that early presentations are not negated by delayed treatment, a virtuous cycle can be established, in which better onset-to-door and door-to-needle times may further improve each other, leading to a higher proportion of stroke patients arriving within the therapeutic time window for acute stroke therapies.

## Toward an Improvement in Onset-to-Door Times

Delayed hospital arrival after acute ischemic stroke is a major factor contributing to low thrombolysis rates. We have reviewed many modifiable factors associated with hospital arrival times, with patient awareness of emergency pathways and the improvement of emergency medical systems being the strongest targets for intervention. Raising the awareness of the varied symptomatology of stroke may also be effective.

Studies on factors associated with prehospital delay after stroke vary widely in their methodology and a more unified approach to this problem and appropriate data collection is warranted. Awareness of stroke represents a key factor, and public education campaigns must be improved and expanded with the view to improve stroke outcomes.

## Author Contributions

Both authors have read and approved the submitted manuscript, and the manuscript has not been published elsewhere in whole or in part. Both authors listed have contributed significantly to the project. Contributions specifically were JW to the conception of the project, interpretation of the data, and critical revision of the manuscript; JP to the acquisition, analysis, and interpretation of the data and drafting of the manuscript.

## Conflict of Interest Statement

The authors declare that the research was conducted in the absence of any commercial or financial relationships that could be construed as a potential conflict of interest. The reviewer SN and handling editor declared their shared affiliation.
